# Proposal and validation of a new classification for trochanteric fractures based on medial buttress and lateral cortical integrity

**DOI:** 10.3389/fsurg.2023.1044941

**Published:** 2023-03-01

**Authors:** Yiran Zhang, Fengshi Zhang, Ci Li, Meng Zhang, Peixun Zhang

**Affiliations:** ^1^ Department of Orthopedics and Trauma, Peking University People’s Hospital, Beijing, China; ^2^ Key Laboratory of Trauma and Neural Regeneration, Ministry of Education, Beijing, China; ^3^ National Center for Trauma Medicine, Beijing, China

**Keywords:** trochanteric fracture, medial buttress, cortical integrity, classification, fracture

## Abstract

**Background:**

Trochanteric fractures usually require surgical treatment. The currently used classification system, such as AO classification, cannot cover all variant types, and is poor in reliability, causing confusion in surgical decision making. This study describes a simple, well-covered, re-liable, accurate, and clinically useful classification.

**Methods:**

We retrospectively reviewed the records of 907 patients with trochanteric fractures treated by us from 1,999 to 2019 and proposed a new classification according to radiographs. Then, 50 records randomly selected in proportion were examined by 10 observers (5 experienced and 5 inexperienced) independently according to AO and the new classification. After a 2-week interval, repeat evaluation was completed. The Kappa coefficient was used to investigate the intra-observer reliability, inter-observer reliability and the agreement between the observers and the “reference standard”.

**Results:**

The new classification system includes 12 types composed of 3 medial groups and 4 lateral groups. According to the medial buttress, the fractures are divided into group I (intact lesser trochanter, adequate but-tress), group II (incomplete lesser trochanter, effective cortical buttress after reduction) and group III (huge defect of the medial cortex). According to the penetration region of the lateral fracture line, the fractures are divided into group A (intact lateral cortex), group B (incomplete lateral cortex), group C (subtrochanteric fractures) and group D (multiple lateral fracture lines). All of the included cases can be classified according to the new classification, of which 34 (3.75%) cases are unclassifiable by the AO classification. Intra-observer: The experienced achieved substantial agreement using both AO [*k* = 0.61 (95% confidence interval 0.46–0.76)] and new classification [*k* = 0.65 (0.55–0.76)]. The inexperienced reached moderate agreement using both AO [*k* = 0.48 (0.33–0.62)] and new classification [*k* = 0.60 (0.50–0.71)]. Inter-observer: The overall reliabilities for AO [*k* = 0.51 (0.49–0.53)] and for new classification [*k* = 0.57 (0.55–0.58)] were both moderate. The agreement between the experienced and the reference standard according to AO [*k* = 0.61 (0.49–0.74)] and new classification [*k* = 0.63 (0.54–0.72)] were both substantial. The agreement between the inexperienced and the reference standard according to AO [*k* = 0.48 (0.45–0.50)] and the new classification [*k* = 0.48 (0.41–0.54)] were both moderate.

**Conclusion:**

Compared with AO classification, our new classification is better in coverage, reliability and accuracy, and has the feasibility of clinical verification and promotion.

## 1. Introduction

Trochanteric fractures occur frequently in the elderly and usually need surgical treatment (1). Image-based classification systems have prognostic value and can assist surgeons in making surgical decisions. An ideal classification system should be simple, reliable, accurate, well-covered and helpful to clinical decision-making. However, the commonly used systems, such as Evans/Evans-Jensen classification and AO classification (2–4), have certain limitations.

In the Evans classification, the medial support which includes lesser trochanter is the key factor affecting the stability of the fractures, but effect of the greater trochanter and the lateral cortex is ignored. In addition, it was developed based on the fracture lines of conservative cases, and thus had limited guiding significance for internal fixation. In the Evans-Jensen classification, these defects have been modified but the reverse fracture is ab-sent. Also, the concept that equating lesser trochanter fractures with no medial support and greater trochanter fractures with no lateral support has been challenged over the years (5, 6).

The AO classification focuses on the description of the fracture morphology, which makes it complex and cumbersome. In previous versions, fractures with incomplete lateral cortex were not systematically described, and sometimes differences between sub-types were too subtle to distinguish, resulting in poor reliability and accuracy in practice (7–12). The 2018 version of AO classification introduced the concept of the lateral wall and used 20.5 mm as the critical thickness to distinguish A1 and A2 group, which was proposed by Hus et al. (13). The thickness of the lateral wall was defined as the distance from a reference point 3 cm below the innominate tubercle of the greater trochanter angled 135° upward to the fracture line on the anteroposterior x-ray (2). This highlighted the importance attached to the integrity of the lateral cortex but the following problems still existed. (1) The correlation between lateral wall thickness and lateral cortical integrity is uncertain. Even if it is thick enough on the anteroposterior x-ray, the lateral strength may still be affected by the coronary fracture lines that often exist in this area (14). Thus, thickness should not be the only basis for judging lateral integrity. Sharma et al. found that lower lateral fracture line penetration site was associated with an increased risk of perioperative lateral rupture (5). Therefore, compared with thickness, perhaps the penetration region of the fracture line has a better indication of the lateral cortical integrity. (2) The measurement is prone to errors. Fractures cause external rotation and the measurement varies with the degree. Sun et al. found that this thickness might include the anterior and posterior cortex, which was an artifact for speculating the strength of the lateral wall (15). (3) The classification is poor in coverage and inaccurate. Elimination of the A2.1 subgroup makes the lateral wall fractures with intact less trochanter unclassifiable. Meanwhile, there are no updates for A3 group. It degrades the accuracy that all kinds of wedge or multifragmentary fractures which may need different surgical treatment can only be classified into subgroup A3.3. Moreover, it is difficult to classify when fracture lines expend to sub-trochanteric area.

Because of this, in order to make up for the shortcomings of the classification system currently used, we developed a new classification for trochanteric fractures based on medial buttress and lateral cortical integrity. The new classification has been preliminarily verified in coverage, reliability, and accuracy, which may have prognostic value and assist surgeons in making surgical decisions.

## 2. Materials and methods

### 2.1. Study design

**Figure 1:**
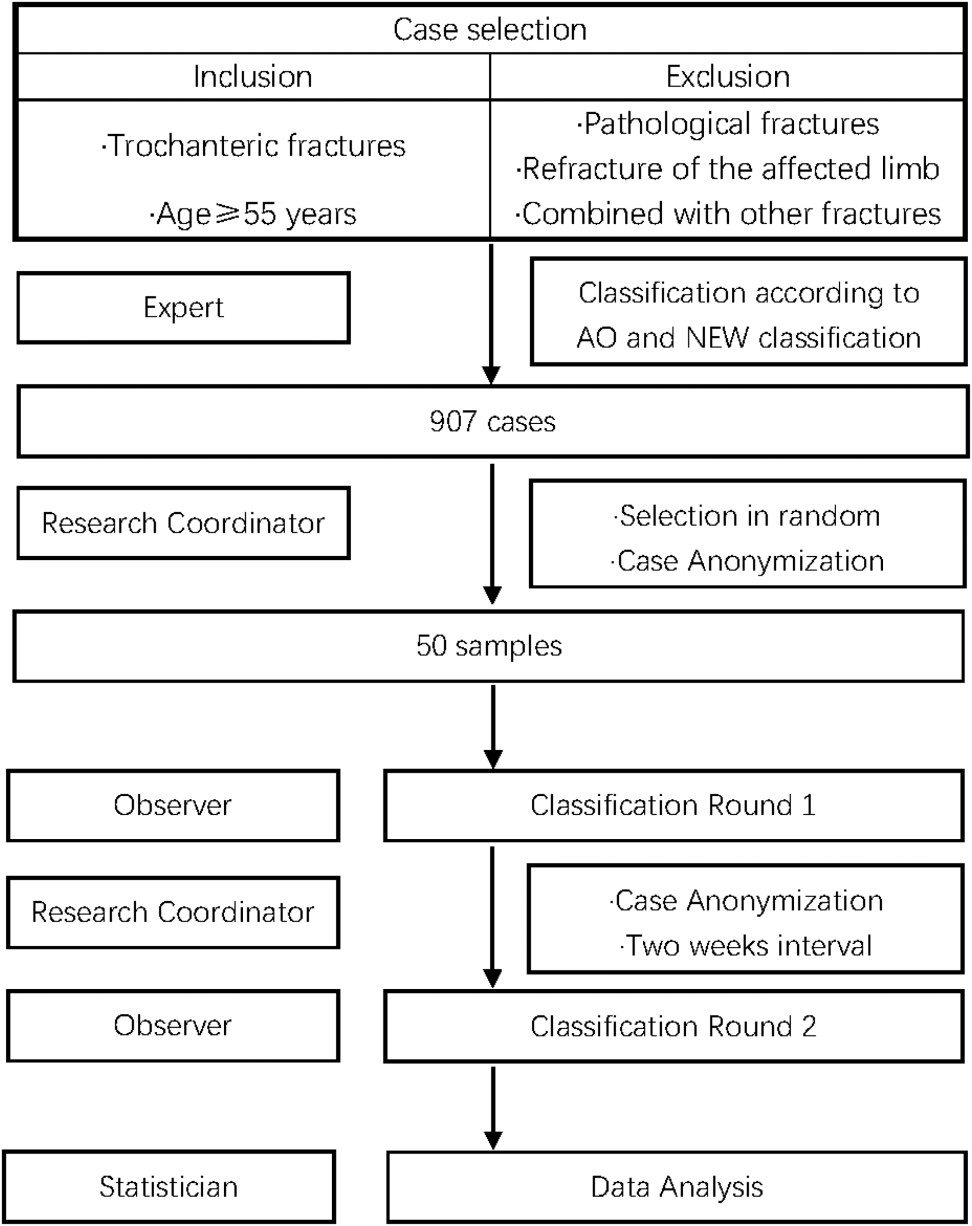
Study design. 50 anteroposterior radiographs of the classified cases were selected in random according to the proportion of AO classification and included: A1.1 (0.77%), A1.2 (25.69%), A1.3 (37.16%), A2.2 (18.96%), A2.3 (1.76%), A3.1 (0.99%), A3.2 (1.21%), A3.3 (9.70%), and AO unclassifiable (3.75%). These selected cases were anonymized by the research coordinator and then classified by 5 orthopedic surgery residents (inexperienced group) and 5 orthopedic traumatologists (experienced group). The observers classified each case individually using the preoperative radiographs only. After a 2-week interval, the same cases were pre-sented in random sequence for repeat evaluation by 1 experienced and 1 inexperienced observer.

We retrospectively reviewed the records (age, gender and preoperative radiographs) for patients with trochanteric fractures treated in our center from January 1999 to March 2019. The distribution of various types in AO and new classification were analyzed by 3 orthopedic experts and finally a consensus opinion was reached through discussion. Ten observers independently used both the AO and the new classification to examine 50 radiographs to evaluate the reliability and accuracy (Figure 1).

All observers received a Microsoft Power Point (PPT) describing the new classification system 2 weeks before the evaluation began. Each observer was familiar with the 2018 AO classification. When fracture patterns were not represented in the classification system, they are classified as “unclassificable”.

The study was conducted in accordance with the Declaration of Helsinki and its later amendments, and approved by Ethics Review Committee of Peking University People's Hospital. Written informed consent was obtained from all the patients involved in the study to participate this study and publish this paper.

### 2.2. New classification system

**Figure 2:**
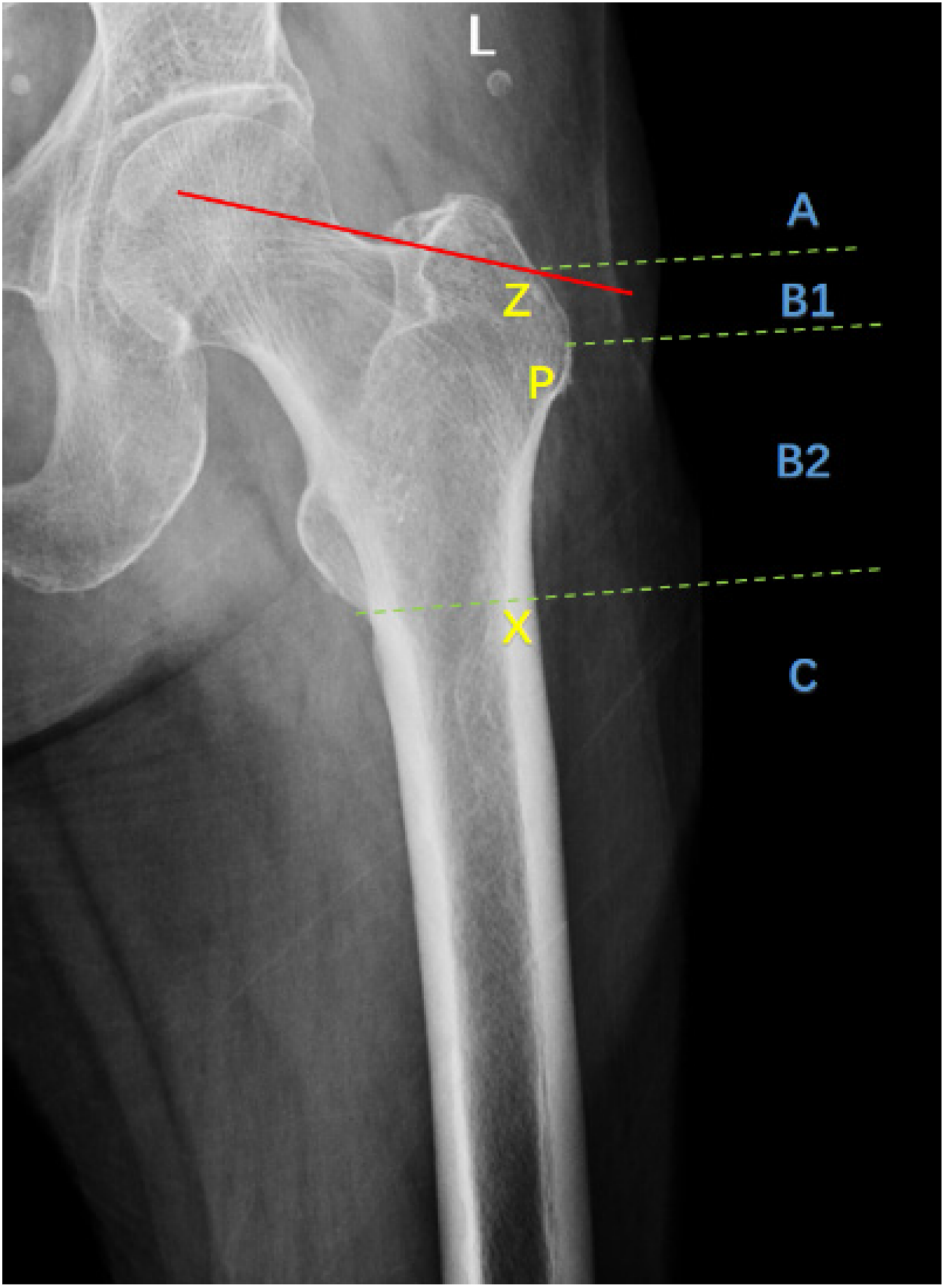
Radiograph showing lateral groups. Z: Make a tangent (red line) to the tension trabecular at the superior cortex of the femoral neck. Z is the intersection of the tangent and the lateral femur (16). P represents the vastus ridge that marks the boundary of the cortex (17). X represents the intersection of the vertical line of the lateral femur passing through the lowest point of the less trochanter. The lateral femur is divided into A/B/C region by points Z/X. Ac-cording to the penetration region of the lateral fracture line, the lateral fractures are divided into three groups: A/B/C. When there are two or more fracture lines, it is group D. Group D can be divided into subgroups by the penetration region of each fracture line, such as D(AC), D(BC), and D(ABC).

The new classification is based on the medial buttress and lateral cortical integrity. It includes 12 types composed of 3 medial groups and 4 lateral groups (Table 1). According to the involvement of the lesser trochanteric and the buttress after reduction, the medial fractures are divided into three groups: I/II/III. The residual cortex as well as medial stability decreases with the increase of the lesser trochanter fragments. According to the penetration region and count of the lateral fracture lines, the lateral fractures are divided into four groups: A/B/C/D. Z: Make a tangent (red line) to the tension trabecular at the superior cortex of the femoral neck. Z is the intersection of the tangent and the lateral femur (16). P represents the vastus ridge that marks the boundary of the cortex (17). X represents the intersection of the vertical line of the lateral femur passing through the lowest point of the less trochanter. The lateral femur is divided into A/B/C region by points Z/X. Ac-cording to the penetration region of the lateral fracture line, the lateral fractures are divided into three groups: A/B/C. When there are two or more fracture lines, it is group D. Group D can be divided into subgroups by the penetration region of each fracture line, such as D(AC), D(BC), and D(ABC). The cortical integrity as well as lateral stability decreases with the descent of the penetration region and the increase of the count (Figure 2).

**Table 1:** The new classification.

Type	Medial group	Lateral group	Medial buttress	Lateral cortical integrity
IA	I	A	Yes: Intact lesser trochanter; Adequate buttress	Yes
IB		B		No
IC		C		No
ID		D		No
IIA	II	A	Yes: Incomplete/Isolated lesser trochanter; Effective cortical buttress after reduction	Yes
IIB		B		No
IIC		C		No
IID		D		No
IIIA	III	A	No: Isolated lesser trochanter with huge cortical fragments; Huge defect of the medial cortex; No cortical contact after reduction	Yes
IIIB		B		No
IIIC		C		No
IIID		D		No

### 2.3. Statistical analysis

The Kappa coefficient was used to investigate the intra-observer reliability. The intra-observer reliability was determined by the first-round results and the second-round results by 1 experienced observer and 1 inexperienced observer.

The Fleiss’ kappa coefficient was used to investigate the inter-observer reliability. The inter-observer reliability was determined by the first-round results by 5 experienced observers and 5 inexperienced observers.

The “reference standard” (18) of both AO and new classification was a consensus opinion reached by 3 orthopedic experts through discussion. We divided the fractures into two categories using AO and new classification: lateral cortex intact (A1.1 to A1.3, Group A) and lateral cortex incomplete (A2.2 to A3.3, Group B/C/D). We calculated the observers’ results and the “reference standard” about whether the lateral cortex is intact and measured the agreement between them to estimate the accuracy.

The reliabilities were graded as described by Landis and Koch (19), with 1 representing perfect, 0.81–1 almost perfect, 0.61–0.8 substantial, 0.41–0.60 moderate, 0.21–0.40 fair, 0–0.20 poor. All analyses were performed with IBM SPSS Statistics (version 26.0).

## 3. Results

### 3.1. Patients

**Figure 3:**
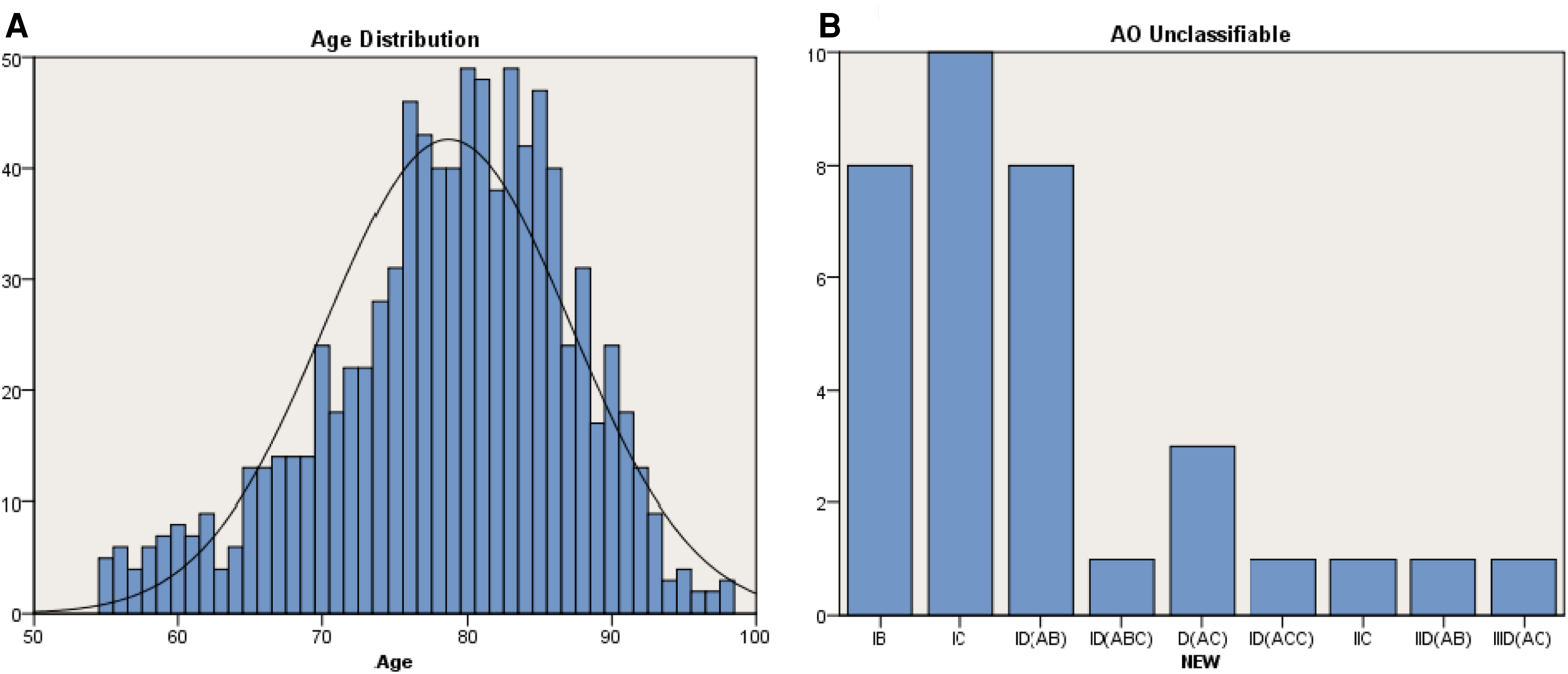
Patients and coverage. (**A**) Age distribution of the study population (*n* = 907, mean age = 78.66 years, males = 356, females = 551). (**B**) Distribution of fractures unclassifiable in AO classification (IB = 8, IC = 10, ID = 13, IIC = 1, IID = 1, IIID = 1).

A total of 907 patients were included in the study with a mean age of 78.66 ± 8.50 years. There were 356 (39.25%) males and 551 (60.75%) females (Figure 3A).

### 3.2. Coverage

All of the included cases can be classified according to the new classification, of which 34 (3.75%) cases were unclassifiable according to the AO classification. These AO unclassifiable cases can be classified in the new classification as IB (8, 23.52%), IC (10, 29.41%), ID (13, 38.24%), IIC (1, 2.94%), IID (1, 2.94%) and IIID (1, 2.94%) (Figure 3B).

### 3.3. Percentages and distribution of fracture types

**Figure 4:**
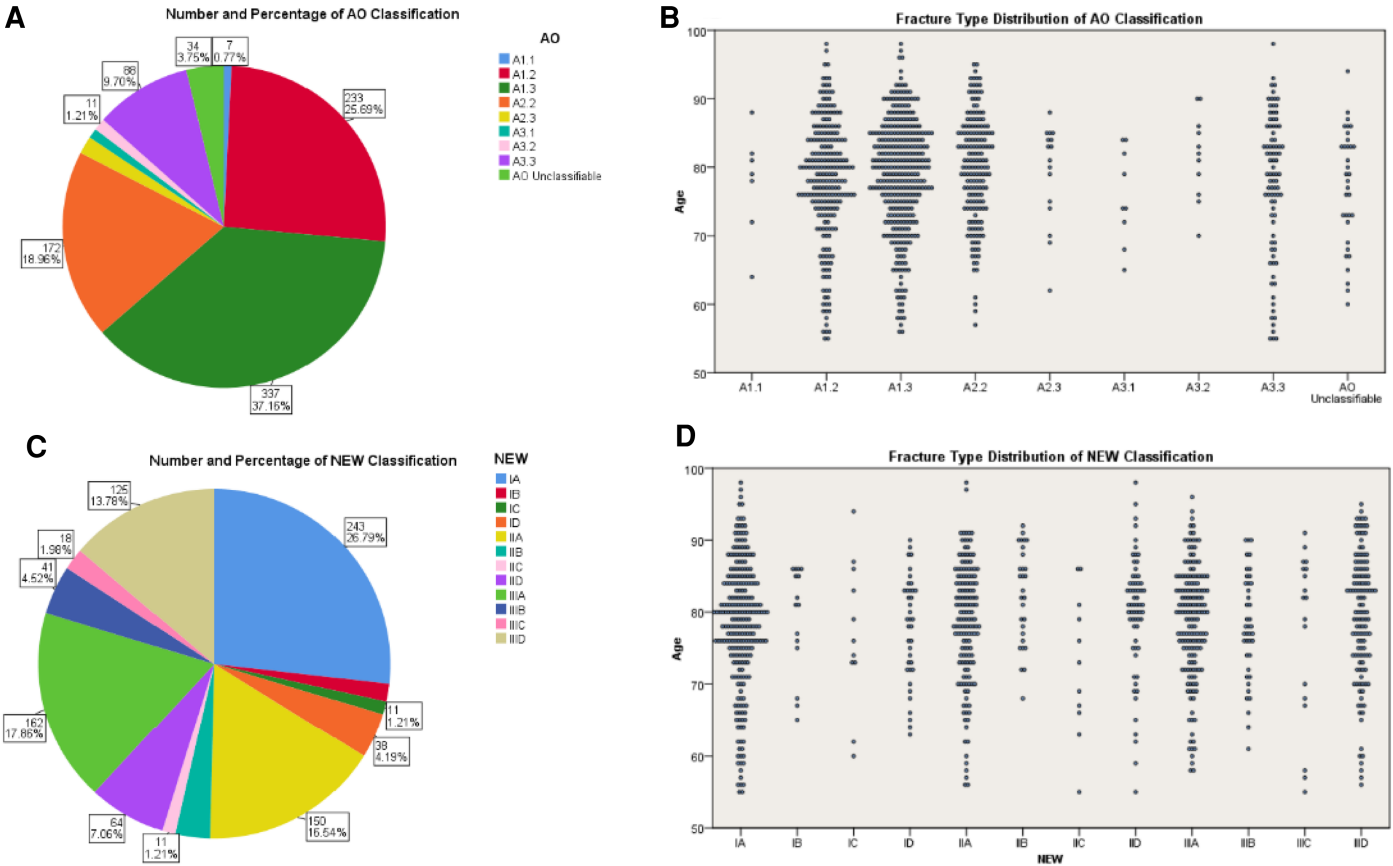
Percentages and distribution of fracture types. (**A**) Number and percentage of the fractures within the study population according to AO classification. (**B**) AO subgroups distribution of fractures in different age groups. (**C**) Number and percentage of the fractures within the study population according to new classification. (**D**) Fracture distribution in different age groups according to new classification.

According to AO classification, the included cases can be divided into A1.1 (7,0.77%), A1.2 (233,25.69%), A1.3 (337,37.16%), A2.2 (172,18.96%), A2.3 (16,1.76%), A3.1 (9,0.99%), A3.2 (11,1.21%), A3.3 (88,9.70%) and AO unclassifiable (34,3.75%) (Figures 4A,B).

According to the new classification, the included cases can be divided into IA (243,26.79%), IB (15,1.65%), IC (11,1.21%), ID (38,4.19%), IIA (150,16.54%), IIB (29,3.20%), IIC (11,1.21%), IID (64,7.06%), IIIA (162,17.86%), IIIB (41,4.52%), IIIC (18,1.98%) and IIID (125,13.78%) (Figures 4C,D).

### 3.4. Reliability and accuracy

#### 3.4.1. Intra-observer reliability

The experienced achieved substantial agreement using both AO [Kappa coefficient = 0.61 (95% confidence interval = 0.46–0.76)] and new classification [0.65 (0.55–0.76)]. The inexperienced reached moderate agreement using both AO [0.48 (0.33–0.62)] and new classification [0.60 (0.50–0.71)] (Table 2).

**Table 2:** Intra-observer reliability.

	Kappa coefficient (95% CI)	
	AO classification	New classification
The experienced	0.61 (0.46–0.76)	0.65 (0.55–0.76)
The inexperienced	0.48 (0.33–0.62)	0.60 (0.50–0.71)

CI, confidence interval.

#### 3.4.2. Inter-observer reliability

The experienced achieved moderate agreement using AO [Kappa coefficient = 0.56 (95% confidence interval = 0.51–0.60)] while substantial agreement using new classification [0.64 (0.61–0.68)]. The inexperienced reached moderate agreement using both AO [0.42 (0.37–0.46)] and new classification [0.49 (0.46–0.52)]. The overall reliabilities for AO [Fleiss’ kappa coefficient = 0.51 (0.49–0.53)] and for new classification [0.57 (0.55–0.58)] were both moderate (Table 3).

**Table 3:** Inter-observer reliability.

	Fleiss’ Kappa coefficient (95% CI)	
	AO classification	New classification
The experienced	0.56 (0.51–0.60)	0.64 (0.61–0.68)
The inexperienced	0.42 (0.37–0.46)	0.49 (0.46–0.52)
Overall	0.51 (0.49–0.53)	0.57 (0.55–0.58)

CI, confidence interval.

#### 3.4.3. Agreement between observers and reference standard

The agreement between the experienced and the reference standard according to AO [mean Kappa coefficient = 0.61 (0.49–0.74)] and new classification [0.63 (0.54–0.72)] were both substantial. The agreement between the inexperienced and the reference standard according to AO [0.48 (0.45–0.50)] and the new classification [0.48 (0.41–0.54)] were both moderate (Table 4).

**Table 4:** Reliability between observer and reference standard.

	Kappa coefficient (95% CI)			
	The experienced		The inexperienced	
	AO classification	New classification	AO classification	New classification
**Observer**				
1	0.79 (0.51–1.06)	0.63 (0.36–0.91)	0.45 (0.17–0.73)	0.51 (0.23–0.79)
2	0.62 (0.35–0.90)	0.52 (0.24–0.79)	0.47 (0.19–0.75)	0.40 (0.12–0.68)
3	0.55 (0.27–0.82)	0.71 (0.43–0.99)	0.49 (0.21–0.77)	0.48 (0.20–0.75)
4	0.58 (0.31–0.86)	0.67 (0.39–0.95)	0.50 (0.22–0.78)	0.48 (0.20–0.76)
5	0.54 (0.26–0.82)	0.64 (0.36–0.91)	0.46 (0.19–0.74)	0.56 (0.28–0.83)
Overall	0.61 (0.49–0.74)	0.63 (0.54–0.72)	0.48 (0.45–0.50)	0.48 (0.41–0.54)

CI, confidence interval.

## 4. Discussion

To make the classification have prognostic value and assist in surgical management, we reviewed the recent researches on the concept of trochanteric fractures classification when developing the new classification. Under normal circumstances, the medial structure bears the main compressive stress of the proximal femur while the lateral structure bears the main tensile stress of trochanteric region (20). When fracture happens, the continuity is interrupted and the stress balance is broken. The ideal treatment is to redistribute stress and restore stability by reduction and internal fixation. The stability after reduction varies with the fracture patterns, and the choice of internal fixation as well as the prognosis will change accordingly. Therefore, the classification of trochanteric fractures should focus on the stability.

Evans proposed that the integrity of posterior medial structure containing the lesser trochanter determined the stability of trochanteric fractures, and the key to restoring stability was to restore the support of the medial cortex (21). This concept has been widely accepted (22–24) and has become an important basis for classifications (25, 26). It was verified by biomechanics that the stability decreased with the increase of the size of lesser trochanter fragment (27) and fixing the fragment could make it more stable after reduction and fixation (28). However, the results of clinical studies have made the importance of lesser trochanteric fragment controversial (6, 29, 30). The medial structure includes the lesser trochanter and its surrounding cortex. We suppose that the key to stability is whether there is adequate residual cortex for medial buttress after reduction, rather than whether there is lesser trochanter fragment. Sharma et al. analyzed 12 cases of fractures with medial defects by CT and suggested that the lesser trochanter fragments could not predict the stability of fractures (5). However, the author also pointed out in these cases, the medial structure except the lesser trochanter was mainly the distal cortex of the head and neck fragment, which had been effectively reduced during the operation. This is consistent with our view that cortex buttress determines the medial stability. Based on that, our new classification divides the medial fractures into three groups.

Another basis for the new classification is the integrity of the lateral cortex. Studies based on extramedullary (17, 31, 32) and intramedullary (33) fixation have shown that the intact lateral cortex plays an important role in postoperative stability. For fractures with incomplete lateral cortex, Sharma et al. found that the risk of perioperative lateral ruptures increased with the descent of the penetration region of lateral fracture lines (5). Bryan et al. stated that for highly unstable trochanteric fractures, the absence of lateral support was the main factor leading to fixation failure (34). On the basis of previous AO classification, Gotfried (31) and Palm et al. (17) divided trochanteric fractures into three groups: intact lateral wall, high risk lateral wall and ruptured lateral wall. The 2018 AO classification also added the assessment of lateral fracture, but the method remains controversial (14, 15). In our new classification, the assessment of lateral fractures is more convenient and intuitive.

**Figure 5:**
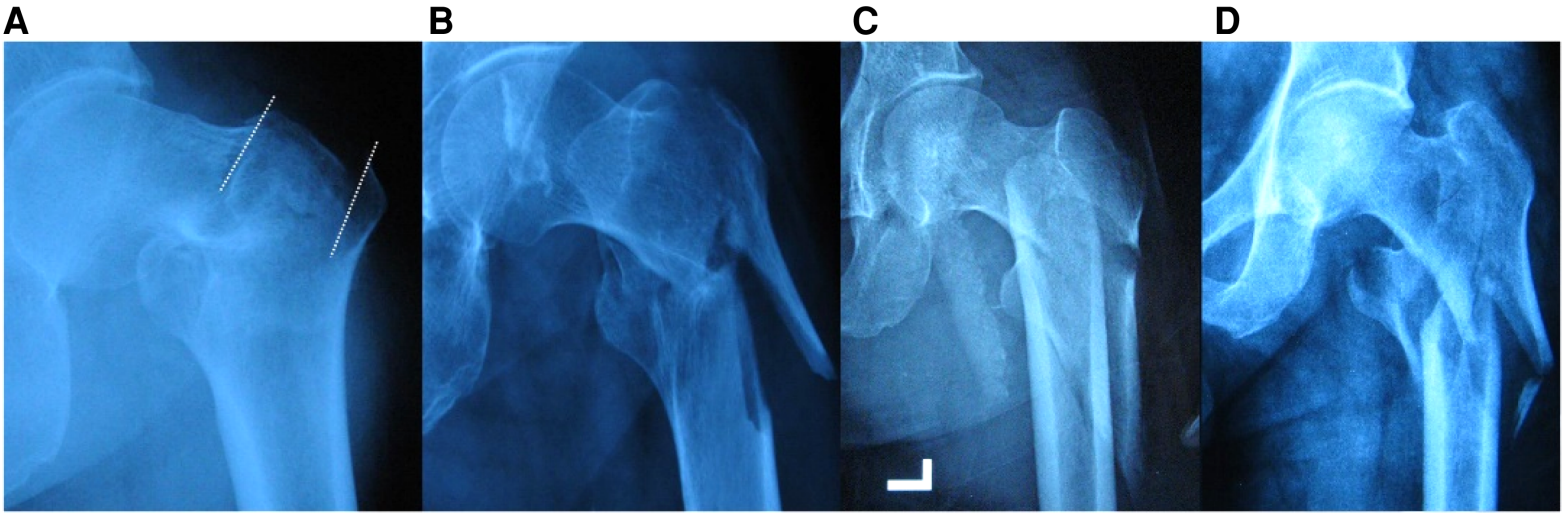
Representative radiographs. (**A**) Type ID(AB) fracture that cannot be classified in AO. It is a fracture with intact medial cortex and two lateral penetrating lines (white dotted line). (**B**) Type ID(AC) fracture that cannot be classified in AO. (**C**) Type ID(BC) fracture that cannot be classified in AO. (**D**) A3.3/IIID(AC) fracture.

Group I and group II are medial stable fractures. In this study, type IA accounted for the highest proportion, 23.79%. This type has adequate medial buttress, high penetration region of lateral fracture line and complete lateral cortex. These elements make it rather stable and similar to subgroup A1.2. Type IB is stable on the medial side, but its lateral stability decreases as the penetration region descends, including lateral high-risk (Figure 2, zone B1) and lateral rupture (Figure 2, zone B2). This type can't be classified in AO classification. Type ID includes fractures with multiple lateral lines penetrating from different regions. There is no description of fractures which only involve the lateral cortex (Figure 5A) in the AO classification. When there are fracture lines in zone C, the subtrochanteric region is involved (Figures 5B,C). Group III are medial unstable fractures with lack of effective buttress. Of this group, type IIIA has the highest proportion, which is 17.85%. This type has intact lateral cortex and is similar to subgroup A1.3. Type IIID ranks second with 13.78%. The fractures included are usually complex and multifragmentary. When the lateral fracture line penetrates from zone B or below (Figure 5D), it is the type with incomplete lateral cortex and lack of medial buttress, which makes its stability the worst.

The extramedullary fixation system represented by Dynamic Hip Screw (DHS) used to be the standard fixation method for trochanteric fractures. But when lateral cortex was involved, the risk of fixation failure increased (17, 31, 32). Under that circumstances, intramedullary fixation is recommended (23). However, even by intramedullary fixation, failure is also likely to happen when the lateral rupture is severe, as the lateral buttress for the head screw is absent (16). One possible solution is to rebuild the lateral structure with plate to restore stability. Hsu et al. proposed a lateral stabilization plate that can reduce the incidence of postoperative fixation failure when combined with extramedullary fixation (35). But the fixation for reconstruction of the lateral structure is not yet popular, and the method as well as its effect needs further study.

Effective medial cortical buttress plays a key role in maintaining postoperative stability and combating varus. Generally speaking, no specific medial management during operation is required for fractures of group I. As for fractures with isolated lesser trochanter like group II, clinicians tend to ensure the effective contact of the medial cortex after reduction, instead of perfect reduction of the fragments. The key point is to put the medial cortex of the proximal fragment in alignment with or in the inner side of the cortex of the distal broken end. This is called positive medial cortical apposition (30). So as to resist varus stress and prevent internal fixation from cutting out. The medial cortical defect of group III is too severe to restore medial cortical contact only by intraoperative reduction. Thus, postoperative weight bearing usually needs delay to avoid rotation and varus. For these fractures, it may be possible to rebuild the medial support structure with bone grafting and/or plate nails in order to restore stability. Ye et al. reported the use of cannulated screws combined with medial support plates for Pauwels 3 vertical unstable femoral neck fractures (36). The follow-up results proved that the healing rate was improved. There are few reports of medial support plates to reconstruct the medial structure of trochanteric fractures. Further study is needed.

Fracture classification evolves dynamically with new and enhanced imaging modalities. Chang et al. proposed a four-by-four sophisticated fracture classification system for the proximal femur trochanteric region (AO/OTA-31A) based on 3D-CT images and accommodated the clinical requirement of the worldwide outbreak of geriatric hip fractures with large amounts of surgical operations (37). In this study, we assessed and compared the new classification and the AO classification in coverage, reliability and accuracy. In terms of coverage, there is no description of fractures which only involve the lateral cortex (Figure 5A) in the AO classification, and when the fracture expends to the subtrochanteric area (Figures 5B,C), it cannot be clearly classified either. These types can be accurately classified in the new classification (Figure 3B), which indicates the new classification is better in coverage.

As for distribution of patients with different ages and types, the fractures in both classification systems were concentrated in the higher age. They were particularly concentrated in A1.2/A1.3/A2.2 according to AO classification while rather uniformly distributed by the new classification. This might be related to accuracy. For example, fractures classified as A1.3 could be type IIA or IIIA. During operation, the former needs medial cortex contacts after reduction, while the latter may need medial reconstruction. In this respect, the new classification is better for clinical decision-making.

Regarding reliability, the new classification is better for the inter-observer reliability of experienced surgeons. The overall reliabilities for AO [Fleiss’ kappa coefficient = 0.51 (0.49–0.53)] and for new classification [0.57 (0.55–0.58)] were both moderate. However, the experienced achieved moderate agreement using AO [Kappa coefficient = 0.56 (95% confidence interval = 0.51–0.60)] while substantial agreement using new classification [0.64 (0.61–0.68)]. In addition, we found according to whether AO classification or new classification, the agreement among the experienced observers was better than that of inexperienced. Some previous research conclusions were consistent with this (38). But a study in-volving 65 observers concluded that clinical experience did not affect the reliability (39). We speculate that this may be related to the observers. Those who participated in that study had a minimum of 11 years of experience, which is much higher than the inexperienced in our study (less than 5 years).

To estimate the accuracy, we calculated the agreement between the observers and the reference standard. Previous studies have proved that the results of classification developers were reliable (40) and were suitable for use as a reference standard when verifying the system (18). The results of surgeons trained by the developers, expert consensus and observer consensus have appeared in previous studies as standard for comparing and verifying (41–45). Therefore, we used the results of developers as reference standard, and estimated the accuracy by comparing it with the observers’ results about the integrity of the lateral cortex. This is mainly because the AO classification has clear criteria for involvement of the lateral side, but no specific description for the medial.

This study had the following limitations. First, it was a single-center retrospective study. All the included patients were admitted by our trauma center only, which might affect the representativeness. But the overall sample size was relatively large, which could weaken the influence of this factor to some extent. Second, it might compromise the results that the intra-observer reliability was represented by one experienced and one inexperienced observer. Third, it was a preliminary study and how this new classification practically influenced the results of treatment was not verified. The following research mainly focuses on how the new classification can guide clinical decision-making.

## 5. Conclusion

We proposed a simple and well-covered classification for trochanteric fractures. By comparing it with AO classification, we initially verified the coverage, reliability and ac-curacy. Studies are required for further assessment of clinical effectiveness and feasibility.

## Data Availability

The original contributions presented in the study are included in the article/Supplementary Material, further inquiries can be directed to the corresponding author.
